# Development of Machine Learning Model to Predict the 5-Year Risk of Starting Biologic Agents in Patients with Inflammatory Bowel Disease (IBD): K-CDM Network Study

**DOI:** 10.3390/jcm9113427

**Published:** 2020-10-26

**Authors:** Youn I Choi, Sung Jin Park, Jun-Won Chung, Kyoung Oh Kim, Jae Hee Cho, Young Jae Kim, Kang Yoon Lee, Kwang Gi Kim, Dong Kyun Park, Yoon Jae Kim

**Affiliations:** 1 Department of Gastroenterology, Gachon University College of Internal Medicine, Gil Medical Center, 405-760 1198 Guwol dong, Namdong-gu, Incheon 21565, Korea; cys7like@hanmail.net (Y.I.C.); drgreen@gilhospital.com (J.-W.C.); kkoimge@gilhospital.com (K.O.K.); jhcho9328@gilhospital.com (J.H.C.); pdk66@gilhospital.com (D.K.P.); 2 Department of Biomedical Engineering, Gachon University College of Medicine, Incheon 21565, Korea; sungjin9999@naver.com (S.J.P.); kimyj10528@gmail.com (Y.J.K.); 3 Department of Computer Engineering, Gachon University, Seongnamdaero, Sugjeong-gu, Seongnam-si, Gyenonggi-do 13306, Korea; keylee@gachon.ac.kr

**Keywords:** machine-learning, IBD, UC, CD

## Abstract

Background: The incidence and global burden of inflammatory bowel disease (IBD) have steadily increased in the past few decades. Improved methods to stratify risk and predict disease-related outcomes are required for IBD. Aim: The aim of this study was to develop and validate a machine learning (ML) model to predict the 5-year risk of starting biologic agents in IBD patients. Method: We applied an ML method to the database of the Korean common data model (K-CDM) network, a data sharing consortium of tertiary centers in Korea, to develop a model to predict the 5-year risk of starting biologic agents in IBD patients. The records analyzed were those of patients diagnosed with IBD between January 2006 and June 2017 at Gil Medical Center (GMC; *n* = 1299) or present in the K-CDM network (*n* = 3286). The ML algorithm was developed to predict 5- year risk of starting biologic agents in IBD patients using data from GMC and externally validated with the K-CDM network database. Result: The ML model for prediction of IBD-related outcomes at 5 years after diagnosis yielded an area under the curve (AUC) of 0.86 (95% CI: 0.82–0.92), in an internal validation study carried out at GMC. The model performed consistently across a range of other datasets, including that of the K-CDM network (AUC = 0.81; 95% CI: 0.80–0.85), in an external validation study. Conclusion: The ML-based prediction model can be used to identify IBD-related outcomes in patients at risk, enabling physicians to perform close follow-up based on the patient’s risk level, estimated through the ML algorithm.

## 1. Introduction

Inflammatory bowel disease (IBD) consists of a spectrum of chronic and progressive inflammatory disorders including Crohn’s disease (CD) and ulcerative colitis (UC) [1,2]. Incidence of IBD markedly increased over the latter part of the 20th century, and the global burden of IBD has steadily increased [1,3,4,5]. According to recent reports from Global Burden of Diseases, Injuries, and Risk Factors Study (GBD) 2017, the age standardized prevalence rate of IBD increased from 79.5 per 10^5^ population in 1990 to 84.3 per 10^5^ population in 2017 globally [6], despite advances in therapy, and hospital admissions [3,6,7,8].

In recognition of aforementioned troubling trend of IBD worldwide, and to decrease disease-related burden, a number of clinical risk factors have been examined to predict outcomes in IBD. To take proper medical diagnosis, and decision has been associated to improve prognosis among IBD patients. Even recent studies have evaluated biomarkers for IBD flare-up [9,10,11], however, most of the factors suggested in previous research have limitations [9,10,11]. Limsrivilai et al. investigated the predictors for high health care utilization among IBD patients as a single center study, and they found that psychiatric illness, use of corticosteroids, use of narcotics, low levels of hemoglobin, and high numbers of IBD related hospitalizations were associated with worsened prognosis for IBD patients [10]. The area under receive operating curves (AUROC) of logistic regression model using aforementioned variables to predict IBD related hospitalization, and emergency department visits were 0.75 and 0.74, respectively [10]. However, the predictive variables they showed are not available at early phases of the disease, before IBD progression. Khan et al. showed that early use of corticosteroids requirement after diagnosis of UC was associated with a more severe long-term course of UC using nationwide cohort11. However, it is likely that early steroid use reflects disease severity, since it is generally recommended that moderately severe and severe IBD patients were treated with corticosteroids.

As for expanding the rationale for the use of the machine learning model is as follows. Machine learning (ML) is a methodology that can examine large datasets to develop prediction models [12,13] [14,15] and is known to have several advantages over traditional statistical approaches14. It has been used in many areas and is on the verge of application in the medical field including disease related outcome prediction, type classification, even epigenomics [13,16,17,18,19,20,21,22,23,24,25,26,27]. However, to date, no ML algorithm has been developed for clinical studies of IBD outcomes, especially in Asian countries.

In this regards, we aimed to develop a risk prediction model of 5-year IBD-related outcomes based on an ML algorithm, internally validated its performance at Gil Medical Center (GMC), and externally validated a large sample derived from the Korean common data model (K-CDM) network.

## 2. Methods

### 2.1. Institutional Ethic Review Board Approval of the Study Design

This study followed the tenets set forth in the Declaration of Helsinki. The protocol used in this study was reviewed and approved by the institutional review boards of the ethics committees of the GMC (IRB approval number of GMC: GA IRB 2018051). All research was performed in accordance with the national guidelines and regulations.

### 2.2. GMC and Korean Common Data Model (K-CDM) Network Database

We used data from GMC and K-CDM network database. GMC and K-CDM network database was developed from Korean Observational Health Data Sciences and Informatics (K-OHDSI) consortium which is an international collaboration that aims to develop data-sharing systems though CDM model applying open-source data analytics to a large number of health databases [28,29]. Each institute of the Korean OHDSI CDM consortium transfers their electronic medical data (EMR) databases to the CDM model of OHDSI consortium. CDM data accuracy of OHDSI CDM database was validated in previous studies [30]. More detailed information on the process of study using OHDSI CDM network database was described in our previous study [31].

### 2.3. Definition of IBD

IBD is listed in the copayment system regulated by the National Health Insurance System (NHIS); all IBD patients should be registered as incurable and rare disease patients. Once a patient is diagnosed as IBD, one of the Rare and Incurable diseases in Korea, by physicians, the patient is entitled for reduced coinsurance rate of medical expenses, regardless if they are hospitalized or outpatient. Therefore, physicians’ diagnosis and registration of IBD in Korea are tightly controlled by the NHIS [32].

In this study, we included IBD patients aged over 18 years, and diagnosed between January 2006 and June 2017 at GMC or listed in the K-CDM network database. The K-CDM network is a data sharing consortium of tertiary centers in Korea, and we used data from the K-CDM network database that were available during the study period. We excluded patients who were (1) aged under 18 years, (2) lacking at least 1 years’ worth of data after the initial IBD diagnosis, or (3) were missing more than 50% of the laboratory values of interest before data imputation.

The reason why we exclude patients without at least 1 year of valid data after initial IBD diagnosis was as follows; it needs enough follow up periods to differentiate inflammatory bowel disease from intestinal tuberculosis. It is not easy to differentiate inflammatory bowel disease from intestinal tuberculosis at initial presentation because of the reasons below. First, in Korea since intestinal tuberculosis is not rare, and initial symptom presentation of either intestinal tuberculosis or inflammatory bowel disease is similar, physicians generally have in mind that when patients are presented with chronic entercolitis, either intestinal tuberculosis, or inflammatory bowel disease might be possible. Second, there is no single conclusive diagnostic tool to differentiate inflammatory bowel disease from intestinal tuberculosis. Diagnostic process of intestinal tuberculosis or inflammatory bowel disease includes comprehensive consideration for clinical manifestations, colonoscopic findings, and histologic examination. When any mycobacterial staining, polymerase chain reaction test (PCR), culture for mycobacterium is positive from the colonic tissue, tentative diagnosis for intestinal tuberculosis is possible. However, only less than 30% of intestinal tuberculosis cases have positive results of aforementioned studies. Therefore, empirical treatment for intestinal tuberculosis is also part for differential diagnosis of intestinal tuberculosis from inflammatory bowel disease. In Korea, generally, more than 80% of intestinal tuberculosis patients react immediately after anti-tuberculosis medication within 2–3 weeks, therefore, empirical 2–3 weeks of anti-tuberculosis treatment could be the part of tentative diagnosis of intestinal tuberculosis from inflammatory bowel disease.

However, in clinical practice, there might still be difficult cases to differential diagnosis inflammatory bowel disease from intestinal tuberculosis even after 2–3 weeks of anti-tuberculosis treatment. Since misdiagnosis of inflammatory bowel disease for intestinal tuberculosis, and vice versa, is critical, and delayed treatment results in worsen disease prognosis, according to Korean Inflammatory bowel disease guidelines, even before confirmative diagnosis for inflammatory bowel disease or intestinal tuberculosis, it is recommended for physicians to promptly start immunosuppressant treatment with continued anti-tuberculosis treatment when patients’ clinical course is not unresponsive to 2 weeks of empirical anti-tuberculosis treatment. Coding accuracy of disease title and code in GMC and K-CDM database were validated in previous studies [29,30].

### 2.4. Definition of Disease Related Outcomes (Starting Biologic Agents in 5 Year after Diagnosis of IBD)

The disease-related outcome was defined as the 5-year risk of starting biologic agents, or first use of biologic agents prescribed for IBD within 5 years of IBD diagnosis. In Korea, physicians are obliged to follow the rules of the NHIS. The use of biologic agents during the first IBD flare up is not covered by the NHIS. The first use of biologic agents in Korean IBD patients should be delayed until they experience two or more flare ups of IBD, or failure of conventional treatments. Therefore, in Korea, starting a biologic agent in IBD patients equates to a “step-up” in the treatment strategy following failure of response to conventional treatment, such as systemic steroids or other immunosuppressants.

We extracted IBD patients’ initial data at diagnosis from the electronic medical records (EMR) of GMC, and from the K-CDM network database, which shares the CDM data among hospitals enrolled in the network.

### 2.5. Predictor Variables

Predictor variables included patients’ age, sex, type of IBD (UC, CD), age at diagnosis, number of previous hospitalizations, last visit to the hospital, corticosteroid prescriptions (including systemic steroid use at initial diagnosis), use of immunosuppressive medication, and laboratory data derived from the complete blood cell count (CBC), chemistries, erythrocyte sedimentation rate (ESR), and high sensitive serum C-reactive protein (hsCRP) level at first symptom presentation or first visit to hospital with symptom. In this study, we used commonly and frequently performed tests in general clinical practice to develop outcome prediction model among IBD patients. We consider the UC and Crohn’s disease subtype as the variables. Sex value and disease variable were handled as categorical variables and other variables were as continuous variables or dummy variables. Total vector length was 29.

These data were obtained from the EMR database. We regarded use of systemic steroids or immunosuppressives at diagnosis as indicators of disease activity at diagnosis, since the Korean IBD treatment guidelines state that systemic steroids or immunosuppressive agents should be used only in patients with moderate or severe disease.

### 2.6. Missing Covariates

Patients missing more than 50% of the laboratory data of interest were excluded from the analysis. In the remaining cases, missing data were substituted by median values of the existing data. In this study, we handled missing data as follows. When the data of specific values were missing and less than 50%, we excluded those cases. When the data were missing and less than 50%, we regarded those values as the average values which were imputed for those columns.

### 2.7. Development of a ML Model

ML methods analyze various features of a dataset and predict outcomes through pattern recognition, whereas conventional logistic regression methods detect linear relations between factors and outcomes. In real-world clinical settings, patients with IBD show varying patterns of disease features that can be used to infer the IBD sub-phenotype, disease-related prognosis, and appropriate drug treatments. ML methods can help us recognize these patterns.

**Figure 1:**
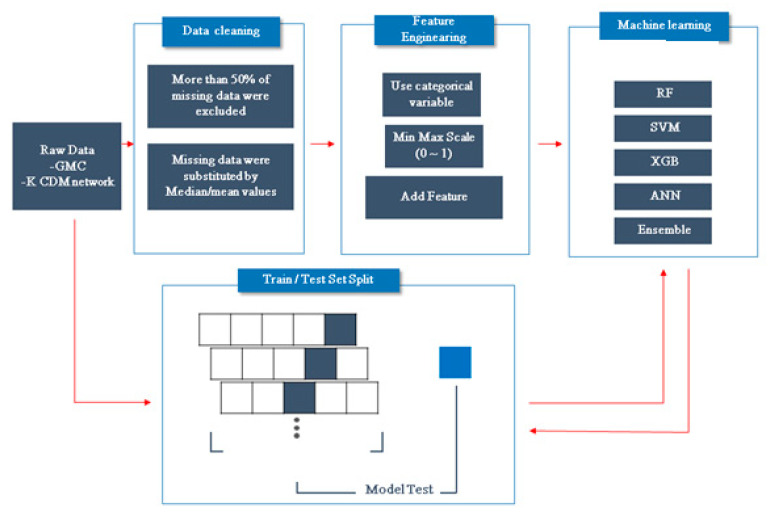
Study flow.

In this study, we used an ML model encompassing support vector machine (SVM) (non-linear model), random forest (RF), XGBoost (XGB), artificial neural network (ANN), and ensemble methods (Figure 1). For patient classification, we used SVM and RF. SVM compares two classes to predict outcomes based on one or more feature vector [33]. RF algorithms constitute a family of classification methods characterized by the combination of several decision trees via recursive partitioning. XGB is a deep learning method based on the majority votes of a set of classification trees. ANNs generate predictions through learning and uncovering new information. An ANN consists of three layers: input, (one or more) hidden, and output layers. We used XGB and ANN as base learners in the ensemble method, which uses diverse subsets of training data.

We divided the patients into training and test sets (ratio = 8:2) in the GMC dataset after then training data set were 5 fold cross validation to find the best optimal parametric values as the heuristic. We developed and trained the ML model for predicting 5-year risk of starting biologic agents using the data obtained at diagnosis of IBD in the training set. Then, we internally validated the predictive performance of the ML algorithm using a test set from the GMC dataset. Finally, we externally validated the ML algorithm using the K-CDM network dataset (the remnant dataset of the K-CDM network after excluding GMC data).

### 2.8. Model Performance

An optimal cutoff was identified to maximize model sensitivity and specificity, and the associated area under the receiver operating characteristic curve (AUROC) was calculated. The ML-based model for predicting risk of starting biologic agents within 5 years after diagnosis, developed from a derivation set of the GMC CDM database, was internally validated using the GMC EMR database and externally validated using data from the K-CDM network. Since the K-CDM network database includes the GMC dataset of IBD patients, we used the remnant dataset of the K-CDM network dataset for external validation, excluding the GMC data.

All analyses were performed using the R (ver. 3.3; R Development Core Team, Vienna, Austria) and Python programs (ver. 2.7; Python Software Foundation, Wilmington, DE, USA). Two-sided *p* values < 0.05 were considered statistically significant.

## 3. Results

### 3.1. GMC and K-CDM Cohorts

IBD patients in the GMC cohort defined as initial IBD diagnosis between 2006 and 2017, and amounted to 1644 cases. After applying inclusion and exclusion criteria, our final cohort consisted of 1299 IBD patients, which we divided into a training set and test set according to an 8:2 ratio. The majority of the patients had UC (763, 58.7%) and were male (782, 60.2%) (Table 1). In total, 135 patients (10.4%) had at least one qualifying biologic agent prescription and were thus deemed to have met the criteria for an IBD-related outcome. The K-CDM cohort, excluding data from GMC, consisted of 1987 IBD patients. A majority of the patients had UC (1060, 53.3%) and were male (1310, 65.9%) (Table 1). In total, 146 patients (8.6%) had at least one qualifying biologic agent prescription and were thus deemed to have met the criteria for an IBD-related outcome.

### 3.2. Internal Validation of an ML-Based Algorithm for Predicting Starting Biologic Agents within 5 Years of IBD Diagnosis

**Figure 2:**
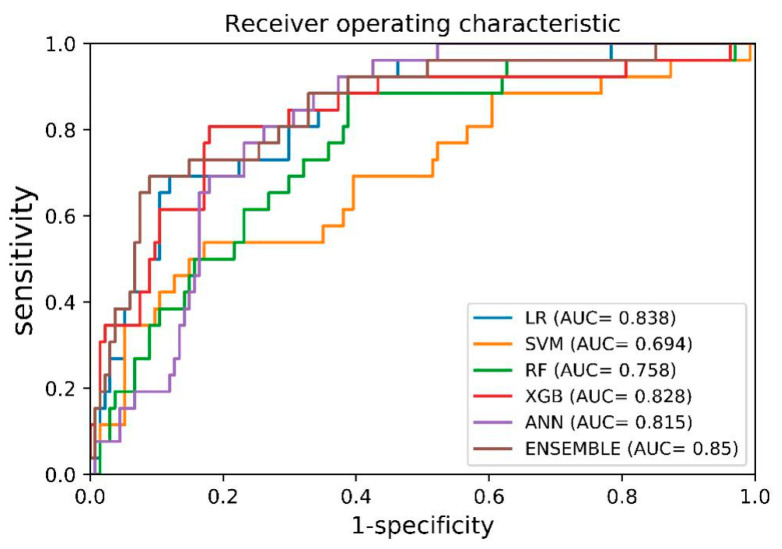
AUROC curves of machine learning (ML) based prediction models for internal validation (using GMC data). Abbreviation: LR, logistic regression; SVM, support vector machine; RF, random forest; XGB, XGBoost; ANN, artificial neural network; AUC, receive operating curve of area under curve.

The AUROC curves for starting biologic agents within 5 years of IBD diagnosis, for the GMC internal validation cohort, are shown in Figure 2. Table 2 shows the model specificity, sensitivity, and AUROC values generated using an ML algorithm based on the SVM, RF, ANN, XGB, and ensemble methods. Among the ML methods, the ensemble method showed the best performance, with an AUROC of 0.85 (95% confidence interval (CI): 0.83–0.87).

### 3.3. External Validation of the ML-Based Algorithm for Predicting Starting Biologic Agents within 5 Years of IBD Diagnosis

**Figure 3:**
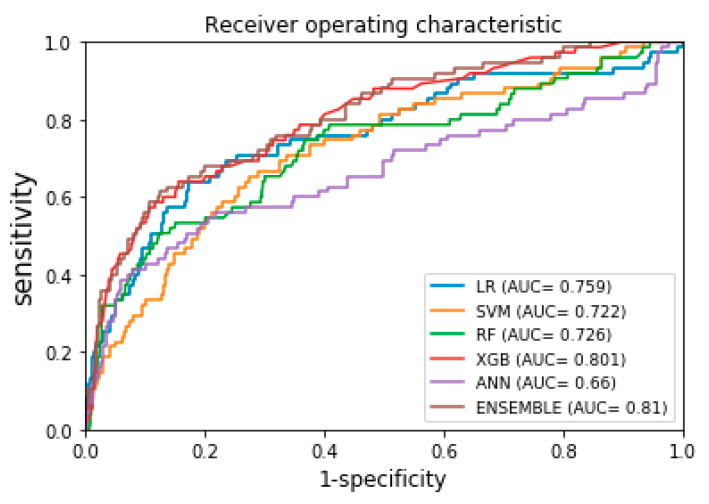
AUROC curves of ML based prediction models for external validation (using k-CDM data). LR, logistic regression; SVM, support vector machine; RF, random forest; XGB, XGBoost; ANN, artificial neural network; AUC, receive operating curve of area under curve.

The AUROC curves for starting biologic agents within 5 years of IBD diagnosis, for the K-CDM network external validation set, are displayed in Figure 3. Table 3 shows the model specificity, sensitivity, and AUROC values generated using the ML algorithm based on SVM, RF, ANN, XGB, and ensemble methods. Among the ML methods, the ensemble method again showed the best performance, with an AUROC of 0.81 (95% CI: 0.79–0.83).

## 4. Discussion

In this study, we developed ML-based algorithms based on SVM, RF, XGB, ANN, and ensemble methods for prediction of an IBD-related outcome (commencement of biologic agents within 5 years of diagnosis) using clinical and laboratory data from GMC and the K-CDM network in Korea. For internal validation, we trained an ML-based prediction model based on the data of 1299 GMC patients and derived an AUROC of 0.85 (95% CI: 0.83–0.87) using the ensemble method. When externally validated using the K-CDM network database, the ML-based ensemble method yielded an AUROC of 0.81 (95% CI: 0.79–0.83). The ML algorithm showed excellent ability to predict the IBD-related outcomes in a Korean population, especially for IBD patients who have experienced at least two flare ups. When comparing the function of prediction model based conventional statistics, ML based prediction model showed better function to predict disease related outcomes (AUROC: 0.76 vs. 0.81).

To our knowledge, this is the first attempt to apply an ML algorithm to predict outcomes in IBD patients, especially those who have experienced two or more flare ups after diagnosis even use of systemic steroid. Since the Korean government provides financial support for IBD patients, via the “Copayment Decreasing Policy for Rare and Intractable Diseases” scheme, the diagnosis of IBD and use of biologics for IBD patients are tightly controlled by the NHIS; biologic treatment for these patients is delayed until at least two flare ups have been experienced after diagnosis. In this study, the commencement of biologics outcome was applicable only to such patients.

As one of the methodologies for managing big data, ML allows computational models to be generated that recognize patterns of data with multiple levels of abstraction [13,14,16,34]. Big data analytics have been shown to be tolerant of poor data quality, but applications are naturally more valid and clinically useful when applied to higher quality data [14]. Moreover, ML is applied to all variables in a large dataset without assuming associations between variables or the predictive power of any particular variable [14]. ML-based prediction models have shown improved performance compared to logistic regression in a variety of clinical settings [14].

There were several attempts to develop ML-based prediction model for the prognosis in IBD patients. Waljee et al. developed an ML-based model for predicting IBD-related outcomes, including hospitalization and outpatient steroid use, among 20,368 Veterans Health Administration patients in the US; the AUROC derived was 0.87 [18]. In another study, also conducted by Waljee et al. based on Veterans Health Administration patient data, a model for predicting clinical remission of 1080 IBD patients on thiopurines generated an AUROC of 0.79 [17]. Both studies used data obtained at diagnosis of IBD, and their designs were similar to that of our study. However, the previous studies used highly specific test and validation datasets (Veterans Health Administration patients); caution is thus necessary when applying the ML algorithm to other groups, even within the US [18]. Furthermore, their investigations were not conducted in a tertiary center, and the study populations might have different characteristics from those derived from tertiary centers. In our study, we used tertiary center data for developing a ML prediction model; to prevent overfitting, the model was validated using the large K-CDM network dataset.

There were several limitations to this study. First, because ML methods have a “black-box” nature [10,13,14], it is difficult to interpret how risk factors interact to affect outcomes. Second, in this study, we enrolled Korean IBD patients who were over 18 years, applying ML algorithm to other ethics, or patients who are under 18 years should be with caution. Third, as the number of potential risk factors increases, the high complexity of the models can cause overfitting; we managed this problem via an external validation study of a large population. Fourth, the ML algorithm was based on a binary classification framework (commencement or non-commencement of biologic agents within 5 years of IBD diagnosis), which can often result in an unbalanced dataset [10]. Thus, we used ensemble learning to construct a balanced dataset and enhance prediction performance. Fifth, missing data is an inevitable issue when using EMR data; therefore, selection bias might have been present in this retrospective study. Sixth, in this study, we did not develop outcome prediction model through machine learning model for UC and CD, respectively, rather we dealt with the disease type of IBD (either UC or CD) as one of the variables for predictive values for final prediction model. Even this methodology was similar to previous research, further investigation should be guaranteed to develop prediction model each for UC and CD, respectively. Seventh, in this study, we especially focused on prediction for IBD patients with ‘step up approaches’ using machine learning methods. Since in Korea, step up approaches have been the main treatment of choice in initial IBD treatment, we could not consider patients with top down approaches even there have been debating between top down approaches and step up approaches in IBD treatment [35,36,37,38]. In this regard, applying our machine learning model to other nations should be done with caution where top down approaches are prominent in IBD treatment. Eighth, in this study, because of non-availability from K-CDM network data, we could not contain values, such as presence of complications (i.e., perianal or fistulizing disease), endoscopy and imaging findings, calprotectin level, extra-intestinal manifestations, and smoking status. Instead, we regarded use of systemic steroids or other immunosuppressive agents at the time of diagnosis as indicative of disease activity. According to Korean IBD treatment guidelines, systemic steroids or other immunosuppressive agents should be prescribed only to patients with moderate or severe diseases, and anti-TNF therapy is typically delayed until the second exacerbation event, or until the patient is unresponsive to, or dependent on, steroids (or immune-suppressants) for at least 3 months after the diagnosis of IBD. Therefore, in Korea, the use of biologic agents is indicative of a poor prognosis. Systemic steroid use at the time of diagnosis is indicative of moderate-to-severe and severe IBD activities. Thus, we used the systemic steroid use at the time of diagnosis as the operational definition of disease severity instead of the UC Mayo score and the CD activity score. Ninth, we did not evaluate whether or not handling missing data policy of patients with more than 50% of missing data were removed from final ML model might affect the performance of developed model or not. Further investigation should be conducted. Tenth, there were differences in durations of diseases before the first biologic agent uses between patients in GMC and those in K-CDM database. The differences in durations of diseases before first biological uses were approximately 40 weeks (1 year). The differences might be resulted from several reasons as below. First, there were differences in UC and CD proportions between GMC and K-CDM database. UC patients were 58.7% (*n* = 763) in GMC database, and 53.3% (*n* = 1060) in K-CDM patients. Second, proportions of disease severity at diagnosis among IBD patients were different between those of GMC and K-CDM database. The proportions of moderately to severe patients of IBD were more prevalent in GMC database. For aforementioned reasons, proportion of duration of disease before the first biologics agent use was different between those of GMC and K-CDM database.

In conclusion, the development and validation of a ML algorithm for prediction of 5-year disease-related outcomes could grant physicians valuable insight into the characteristics of high-risk patients and allow early clinical intervention for IBD, with the aim of reducing disease-related outcomes. Further studies are needed to optimize the accuracy of our algorithm in other populations.

## Figures and Tables

**Table 1:** Demographic characteristics of inflammatory bowel disease (IBD) patients in Gil Medical Center (GMC) and the Korean common data model (K-CDM).

	Derivation and Internal Validation Set from GMC Database (*n* = 1299)	External Validation Set from K-CDM Database (*n* = 1987)
Demographic features and follow-up		
Duration of follow-up, weeks	152.0 ± 157.6	183.0 ± 198.6
Males, *n* (%)	782 (60.2%)	1310 (65.9%)
Age, years	44.3 ± 19.3	45.8 ± 17.7
IBD subtype		
Ulcerative colitis	763 (58.7%)	1060 (53.3%)
Crohn’s disease	536 (41.3%)	927 (46.7%)
Disease behavior at diagnosis		
Age at diagnosis	36.5 ± 18.3	36.7 ± 16.7
Phenotype of IBD		
Systemic steroid use at diagnosis, *n* (%)	712 (54.8%)	733 (36.9%)
IBD related outcome (biologic agent)		
Commencement of Biologic agents, *n* (%)	135 (10.4%)	146 (7.3%)
Ulcerative colitis, *n*	65	70
Crohn’s disease, *n*	70	76
Duration of disease before the first biologic agent use (mean ±SD)(week)	117.3 ± 18.5	232.5 ± 22.2
Laboratory data(at first visit to hospital with symptom)		
Hemoglobin (g/dL)	12.9 ± 2.2	13.1 ± 2.1
Hematocrit (%)	38.7 ± 5.6	38.9 ± 5.6
White blood cell count (10^3^/㎕)	8.3 ± 3.6	8.1 ± 3.5
Serum total bilirubin (mg/dL)	0.7 ± 0.5	0.7 ± 0.5
Serum protein (g/dL)	7.8 ± 15.3	8.3 ± 19.9
Serum albumin (g/dL)	4.1 ± 0.6	5.1 ± 0.5
Serum BUN (mg/dL)	12.7 ± 5.9	12.9 ± 5.0
Serum creatinine (mg/dL)	1.0 ± 3.7	1.0 ± 4.8
Serum hsCRP (mg/L) †	2.1 ± 3.8	1.8 ± 3.6
Serum uric acid (mg/dL)	5.2 ± 1.6	4.9 ± 1.5
Serum cholesterol (mg/dL)	161.8 ± 41.0	170.3 ± 42.0

Abbreviation: IBD, inflammatory bowel disease; GMC, Gil medical center; K-CDM, Korean common data model network; IQR, interquartile range; SD, standard deviation; BUN, blood urea nitrogen; CRP, C-reactive protein; † The reference range for hsCRP was 0–0.5 mg/L.

**Table 2:** Internal Validation of Machine Learning Based Prediction Model Using GMC Data.

Model	Sensitivity (%)	Specificity (%)	AUROC (%)
LR	0.77	0.69	0.83
SVM	0.70	0.65	0.76
RF	0.62	0.58	0.69
ANN	0.77	0.73	0.82
XGB	0.82	0.77	0.83
Ensemble	0.75	0.73	0.85

Abbreviation: LR, logistic regression (conventional statistics); RF, random forest; SVM, support vector machine; ANN, artificial neural network, XGB, XGBoost; AUROC, area under curve receive operating curve.

**Table 3:** External validation of machine learning based prediction model using K-CDM data.

Model	Sensitivity (%)	Specificity (%)	AUROC (%)
LR	0.74	0.69	0.76
SVM	0.68	0.68	0.72
RF	0.67	0.67	0.73
ANN	0.61	0.60	0.66
XGB	0.70	0.71	0.80
Ensemble	0.71	0.71	0.81

Abbreviation: LR, logistic regression (conventional statistics); RF, random forest; SVM, support vector machine; ANN, artificial neural network, XGB, XGBoost; AUROC, area under curve receive operating curve.
